# 757. Evaluating Use of the New ICD-10 Codes for *Clostridioides difficile* Infection in the Premier U.S. Hospital Discharge Database

**DOI:** 10.1093/ofid/ofab466.954

**Published:** 2021-12-04

**Authors:** Abhishek Deshpande, Yiyun Chen, Eugenia Boye-Codjoe, Engels N Obi

**Affiliations:** 1 Cleveland Clinic, Cleveland, Ohio; 2 Merck & Co., Inc, Rahway, New Jersey; 3 Merck & Co., Rahway, New Jersey

## Abstract

**Background:**

The single ICD-10 code for Clostridioides difficile infection (CDI) A04.7 was replaced in Oct 2017 with two codes delineating “recurrent CDI” (rCDI, A04.71) and “nonrecurrent CDI” (nrCDI, A04.72). This study aims to evaluate and validate use of the new ICD-10 codes for CDI among inpatient encounters at hospitals contributing to the Premier Healthcare Database (PHD).

**Methods:**

This retrospective study included inpatient encounters with a CDI-related ICD code between Oct 2016-May 2019 in the PHD. Trends in CDI-related ICD coding were examined pre- and post- the Oct 2017 code update. Post Oct 2017, CDI-related inpatient encounters were characterized by clinical, facility, and provider variables, and whether coding was concordant or discordant to the 2017 IDSA guidelines ‘within 60-days (2 months) from index CDI episode’ time window for capturing rCDI. Multivariable regression examined variables associated with concordant coding.

**Results:**

There was widespread adoption of the new CDI codes across hospitals in the PHD in Oct 2017. Post-Oct 2017, a total of 21,446 CDI-related encounters met sample selection criteria. About 67% of rCDI encounters and 25% of nrCDI encounters were coded concordantly. In the overall sample, the rCDI vs. nrCDI-coded encounters (p< 0.05) had higher proportions with emergency room admission, admitted by a gastroenterologist or infectious disease specialist, and receiving fidaxomicin, bezlotoxumab or FMT. Trends in inpatient characteristics for rCDI vs. nrCDI-coded encounters did not differ by coding concordance status. In regression analyses, encounters coded concordantly were significantly more likely to be for rCDI (OR 5.67), a non-elective admission (OR 1.17-1.42), or prescribed fidaxomicin (OR 1.11), or FMT (OR 1.29).

Encounter Frequency

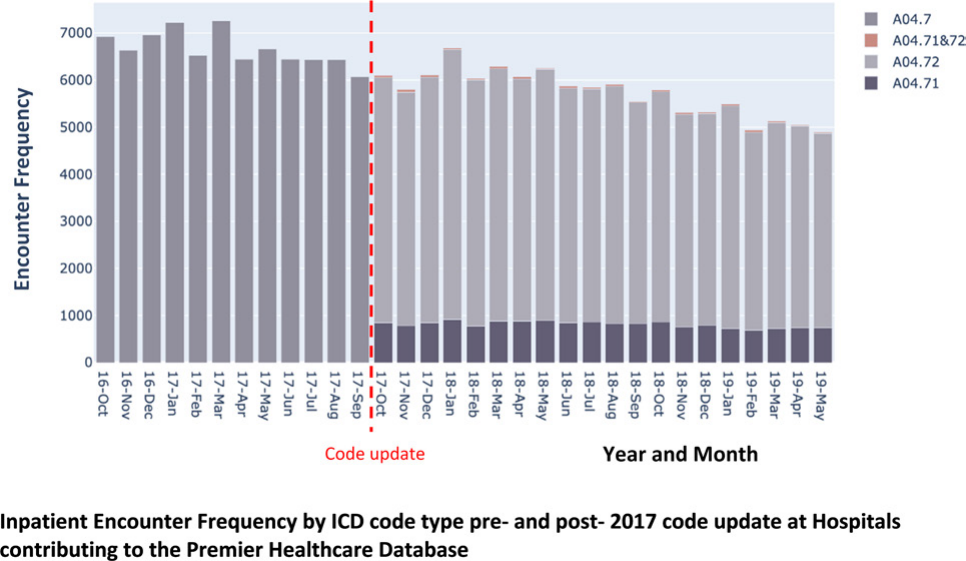

Frequency Table

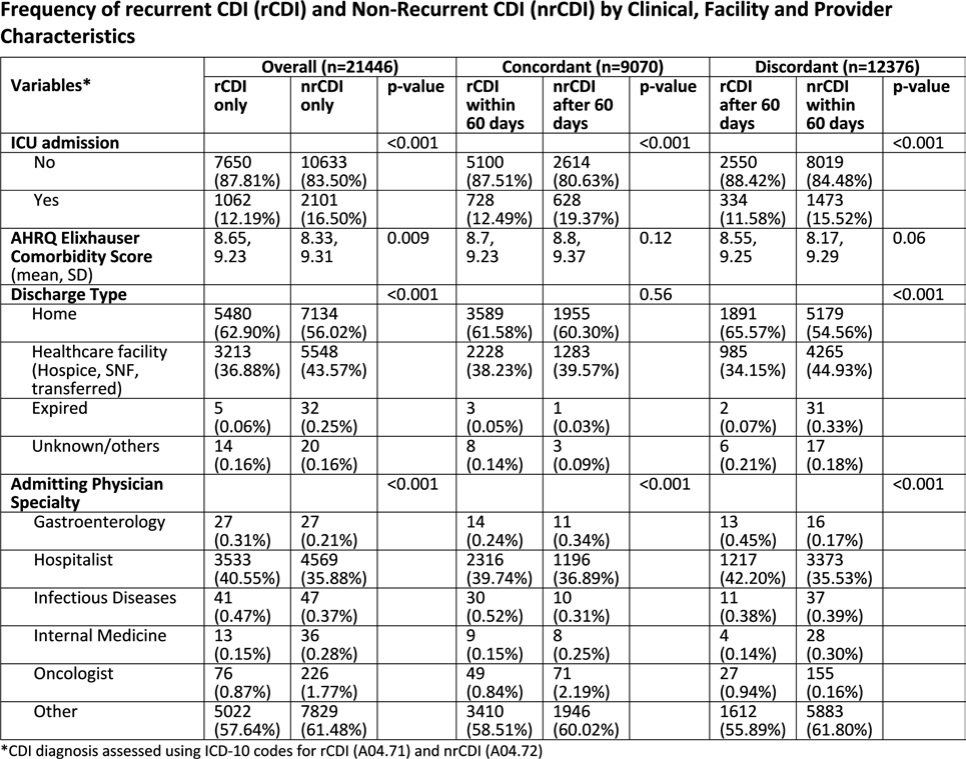

Resource and Cost Table

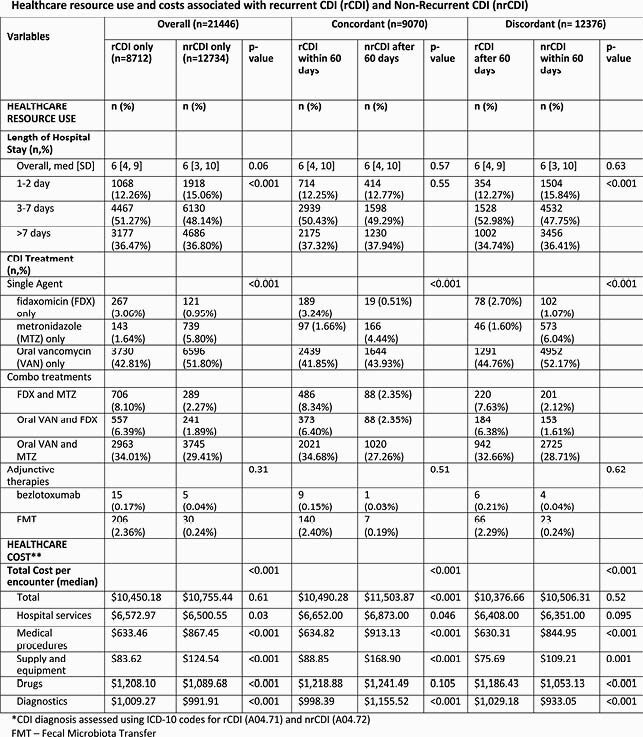

**Conclusion:**

There was no delay in transition to the new CDI-related ICD codes across hospitals in the PHD. Important for disease management, drug treatment trends for encounters coded as rCDI vs. nrCDI were consistent with guideline-recommendations for CDI. Coding concordance status based on the IDSA 60-day time window for identifying rCDI did not affect direction of observed trends in descriptive analyses, suggesting that other validation methods maybe needed.

Regression Table

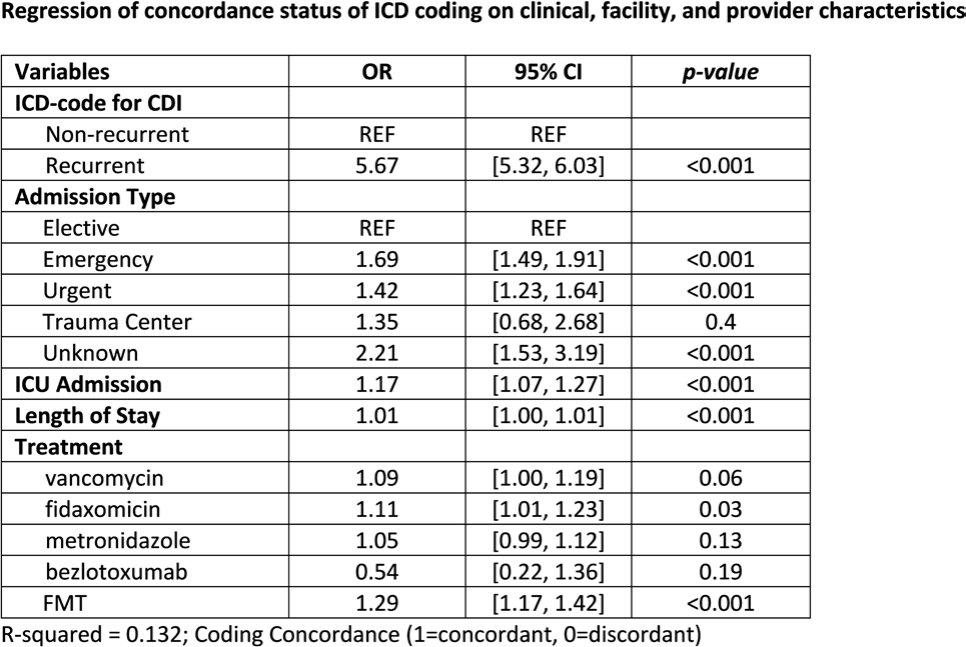

**Disclosures:**

**Abhishek Deshpande, MD, PhD**, **Merck & Co., Inc** (Consultant, Shareholder)**The Clorox Company** (Grant/Research Support) **Yiyun Chen, PhD**, **Merck & Co., Inc** (Employee) **Eugenia Boye-Codjoe, MPH**, **Merck & Co., Inc** (Employee) **Engels N. Obi, PhD**, **Merck & Co.** (Employee, Shareholder)

